# Electroantennogram response of the parasitoid, *Microplitis croceipes* to host-related odors: The discrepancy between relative abundance and level of antennal responses to volatile compound

**DOI:** 10.12688/f1000research.10104.2

**Published:** 2017-03-09

**Authors:** Tolulope Morawo, Matthew Burrows, Henry Fadamiro

**Affiliations:** 1 Department of Entomology & Plant Pathology, Auburn University, Auburn, USA; 2 Division of Parasitic Diseases and Malaria- Entomology, Centers for Disease Control and Prevention, Atlanta, USA

**Keywords:** Braconidae, endoparasitoid, Heliothis virescens, cotton plant

## Abstract

Herbivores emit volatile organic compounds (VOCs) after feeding on plants. Parasitoids exploit these VOCs as odor cues to locate their hosts. In nature, host-related odors are emitted as blends of various compounds occurring in different proportions, and minor blend components can sometimes have profound effects on parasitoid responses. In a previous related study, we identified and quantified VOCs emitted by cotton plant-fed *Heliothis virescens *(Lepidoptera: Noctuidae) larvae, an herbivore host of the parasitoid *Microplitis croceipes* (Hymenoptera: Braconidae). In the present study, the olfactory response of female *M*. *croceipes* to synthetic versions of 15 previously identified compounds was tested in electroantennogram (EAG) bioassays. Using *M. croceipes* as a model species, we further asked the question: does the relative abundance of a volatile compound match the level of antennal response in parasitoids? Female *M. croceipes* showed varying EAG responses to test compounds, indicating different levels of bioactivity in the insect antenna. Eight compounds, including decanal, 1-octen-3-ol, 3-octanone, 2-ethylhexanol, tridecane, tetradecane, α-farnesene and bisabolene, elicited EAG responses above or equal to the 50 ^th^ percentile rank of all responses. Interestingly, decanal, which represented only 1% of the total amount of odors emitted by cotton-fed hosts, elicited the highest (0.82 mV) EAG response in parasitoids. On the other hand, ( *E*)-β-caryophyllene, the most abundant (29%) blend component, elicited a relatively low (0.17 mV) EAG response. The results suggest that EAG response to host-related volatiles in parasitoids is probably more influenced by the ecological relevance or functional role of the compound in the blend, rather than its relative abundance.

## Introduction

Infested plants emit volatile organic compounds (VOCs) as an indirect defense against herbivore damage ^1, 2^. Informative volatile cues used by parasitoids for host location can be emitted by plants infested with herbivores ^1, 2^ or emitted by herbivores that fed on plants ^3, 4^. Although plant volatiles may initially lead parasitoids to the host patch, herbivore host-specific odors are important short-range cues used in the later stages of host location ^5^. The specific mechanism by which plant-fed host larvae emit these volatiles is not fully understood. However, it is evident that parasitoids use these plant-associated VOCs in the host location process ^5^. Such odor cues are usually released as a blend of various compounds in nature. Consequently, differentiating useful cues from ecologically irrelevant odors can be challenging for foraging parasitoids. Therefore, it is expected that antennal sensitivity of parasitoids will vary in response to different compounds. Antenna sensitivity in insects can be measured with electroantennogram (EAG) recording. EAG measures the summed activity of olfactory receptor neurons in the antenna and indicates the level of biological activity elicited by various compounds.

*Microplitis croceipes* (Hymenoptera: Braconidae) is an endoparasitoid of *Heliothis virescens* (Lepidoptera: Noctuidae), which is an important pest of cotton plant. In a previous related study ^5^, female *M. croceipes* showed attraction to the odor blend emitted by cotton-fed *H. virescens* larvae in Y-tube olfactometer bioassays ^5^. The blend components were identified and quantified using gas chromatography-mass spectrometry (GC/MS). Furthermore, the compounds in the attractive blend occurred in varying proportions ( Table 1). However, the relative abundance of a blend component does not necessarily indicate its relevance to resource location in insects ^6^. In the present study, olfactory response of *M*. *croceipes* to synthetic versions of 15 previously identified compounds was tested in EAG bioassays. Comparing EAG results in the present study and GC/MS analyses in a previous study ^5^, we indicated the discrepancy between relative abundance of a volatile blend component and the level of antennal response in parasitoids.

**Table 1.** Composition of headspace volatile organic compounds emitted by cotton-fed *Heliothis virescens* larvae. This table was modified from Morawo and Fadamiro (doi: 10.1007/s10886-016-0779-7) ^5^, with permission from the authors.

ID ^1^	Compound	Relative abundance (%)	Chemical category
1	α-Pinene	15.1	Monoterpene
2	β-Pinene	1.6	Monoterpene
3	1-Octen-3-ol	1.4	Alcohol
4	3-Octanone	0.8	Ketone
5	Myrcene	2.7	Monoterpene
6	*Unknown* ^2^	1.2	-
7	Limonene	9.1	Monoterpene
8	2-Ethylhexanol	2.2	Alcohol
9	Decanal	1.0	Aldehyde
10	Tridecane	6.2	Alkane
11	Tetradecane	2.4	Alkane
12	( *E*)-β-Caryophyllene	29.2	Sesquiterpene
13	α-Bergamotene ^2^	0.7	Sesquiterpene
14	α-Humulene	6.5	Sesquiterpene
15	α-Farnesene	0.8	Sesquiterpene
16	Bisabolene	8.6	Sesquiterpene
17	α-Bisabolol	7.9	Sesquiterpene

^1^In order of elution during gas chromatography. ^2^Compounds that were not tested in the present study.

## Methods and materials

### Insects

*Microplitis croceipes* was reared on 2 ^nd^–3 ^rd^ instar larvae of *Heliothis virescens*. The larvae of *H. virescens* used to rear *M. croceipes* were fed pinto bean artificial diet. Thus, parasitoids had no experience with plant odors. Adult wasps were supplied with 10% sugar water upon emergence in our laboratory at Entomology & Plant Pathology Department, Auburn University. For more details about rearing protocol, see Lewis and Burton ^7^. Female parasitoids used for EAG bioassays were 2–3 days-old, presumed mated (after at least 24 h of interaction with males), and inexperienced with oviposition or plant material. The general rearing conditions for all insects were 25±1 °C, 75±5 % relative humidity and 14:10 h (light:dark) photoperiod.

### EAG recording

EAG responses of *M. croceipes* to 15 synthetic compounds ( Table 1), previously identified in the headspace of cotton-fed *H. virescens* larvae ^5^, were recorded according to the method described by Ngumbi *et al.* ^8^ with modifications. Two compounds, α-bergamotene (not commercially available) and an unidentified compound reported in the previous study ^5^ were not tested in the present study. α-Pinene, β-pinene, myrcene, limonene, 2-ethylhexanol, tridecane, ( *E*)-β-caryophyllene, α-humulene, α-farnesene and α-bisabolol with purity 95–99% were purchased from Sigma-Aldrich ^®^ (St. Louis, MO, USA). 1-Octen-3-ol, 3-octanone, decanal, tetradecane and bisabolene with purity 96–99% were purchased from Alfa Aesar ^®^ (Ward Hill, MA, USA). Test compounds were formulated in hexane at 0.1 μg/μl and delivered onto Whatman ^®^No.1 filter paper strips at an optimum dose of 1 µg. Mass/volume concentration was used to correct for differences in purity of synthetic compounds. The dose was selected as ecologically relevant based on GC/MS analyses results of total amount of volatiles emitted by cotton-fed *H. virescens* larvae ^5^. Impregnated filter papers were placed inside glass Pasteur pipettes and stimulus was introduced as 0.2 s odor puffs. A glass capillary reference electrode filled with 0.1 M KCl was attached to the back of the wasp head, and a similar recording electrode was connected to the excised tip of the wasp antenna. The analog signal was detected through a probe and processed with a data acquisition controller (IDAC-4, Syntech, The Netherlands). Data was assessed using EAG 2000 software (Syntech, The Netherlands). EAG responses to the 15 compounds and control (hexane) were sequentially recorded for each of 15 insect replicates. Each compound was presented at positions 1 through 15 across replicates to minimize positional bias. For instance, 1-octen-3-ol and 3-octanone were introduced to the first insect as the 3 ^rd^ and 4 ^th^ compounds, respectively, but introduced to the second insect as the 4 ^th^ and 5 ^th^ compounds, respectively.

### Data analyses

Differences in absolute EAG values (EAG response to compound minus response to solvent control) of synthetic compounds were analyzed using the *Kruskal-Wallis* test, followed by *Sidak’s* multiple comparison test. The relationship between EAG response and relative abundance was analyzed with *Proc Corr* (correlation) procedure in SAS. All analyses were performed in SAS v9.2 (SAS Institute Inc., Cary, NC, USA) with P=0.05 level of significance.

## Results

**Figure 1.**
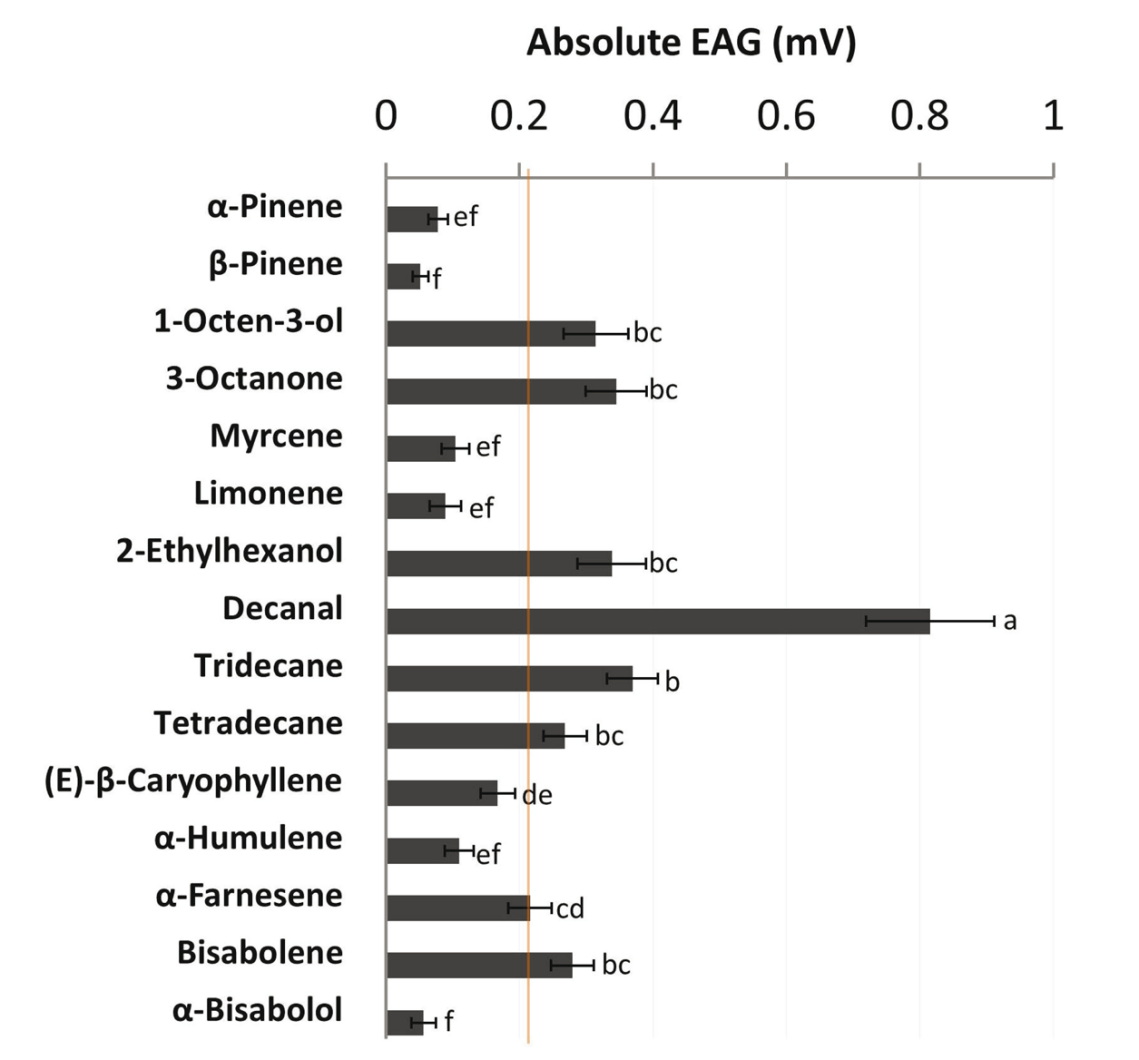
EAG responses of *Microplitis croceipes* to synthetic compounds. Mean absolute Electroantennogram (EAG) responses (mV ± SEM; *N* = 15) of female *Microplitis croceipes* to 15 volatile compounds identified in the headspace of cotton-fed *Heliothis virescens* larvae ^5^. Synthetic compounds were formulated in hexane (solvent control) and tested at an optimum dose of 1 μg. Orange line indicates the arbitrary response threshold of 0.22 mV (50 ^th^ percentile rank). Bars with no letters in common are significantly different ( *P*<0.05; *Kruskal-Wallis* test followed by *Sidak’s* multiple comparison test).

Female *M. croceipes* showed varying EAG responses to test compounds (range: 0.05–0.82 mV; Figure 1). Decanal elicited the highest EAG response (0.82 mV; *χ2* = 134.13; *df* = 14; *P<*0.0001), while β-pinene elicited the lowest response (0.05 mV) in parasitoids. Decanal, tridecane, 3-octanone, 2-ethylhexanol, 1-octen-3-ol, bisabolene, tetradecane and α-farnesene elicited EAG responses ≥0.22 mV (50 ^th^ percentile rank). Four of the top bioactive compounds: decanal, 3-octanone, 1-octen-3-ol and 2-ethylhexanol were emitted in quantities ≤2.2% of the total blend ( Table 1). On the other hand, ( *E*)-β-caryophyllene, the most abundant (29.2% of total blend) component, elicited a relatively low EAG response (0.17 mV) in parasitoids ( Figure 1). However, the negative correlation between EAG response and relative abundance of compounds was not statistically significant (r = -0.33; *N* = 15; *P=*0.23).

EAG responses of Microplitis croceipes to synthetic compounds and correlation with relative abundance of compounds Click here for additional data file. Copyright: © 2017 Morawo T et al. 2017 Data associated with the article are available under the terms of the Creative Commons Zero "No rights reserved" data waiver (CC0 1.0 Public domain dedication).

## Discussion

EAG responses of *Micropiltis croceipes* in the present study indicated variation in biological activity elicited by test compounds at the peripheral level, and revealed a discrepancy between relative abundance and level of antennal responses in parasitoids. High EAG response elicited by decanal in *M. croceipes* agrees with previous reports on olfactory responses of the parasitoids, *Microplitis mediator* ^9^ and *Bracon hebetor* ^10^. Furthermore, decanal is a key attractant for host-seeking *M. croceipes* ^5^. Although compounds are emitted in different quantities in natural blends, minor components can have a profound effect on resource location in parasitoids ^6, 11^. Interestingly, decanal constituted only 1% of the total blend emitted by cotton-fed *H. virescens* ^5^, but elicited the highest EAG response in *M. croceipes*, supporting the “little peaks-big effects” concept ^6^. On the other hand, ( *E*)-β-caryophyllene, the most abundant blend component, elicited a relatively low EAG response in parasitoids.

Therefore, it is more likely that the ecological relevance of a compound, rather than its relative abundance determines the level of olfactory response in foraging insects. For instance, small amounts of isothiocyanates in the volatile blend of brassica plants serve as host location cues for parasitoids of brassica herbivores ^12, 13^. More importantly, blend components act in concert to provide parasitoids with complete information ^14^. Consequently, certain compounds function as background odors to enhance detectability (olfactory contrast) of other attractive components in a blend ^12, 15^. It is possible that ( *E*)-β-caryophyllene serves as a background odor in the blend emitted by cotton-fed *H. virescens*. Finally, it should be noted that while EAG measures the level of bioactivity, behavioral bioassays are usually needed to establish the functional role of various compounds ^5, 16^. In addition, several species of parasitoids can be conditioned to respond to diverse odor cues, regardless of the relevance of such odor cues to their ecology.

## Data availability

The data referenced by this article are under copyright with the following copyright statement: Copyright: © 2017 Morawo T et al.

Data associated with the article are available under the terms of the Creative Commons Zero "No rights reserved" data waiver (CC0 1.0 Public domain dedication). 

**Dataset 1. EAG responses of *Microplitis croceipes* to synthetic compounds and correlation with relative abundance of compounds.** Electroantennogram (EAG) data shows actual EAG response readouts to different compounds for 15 insect replicates. Absolute EAG value for each compound in a replicate can be obtained by deducting the average of two controls (Control 1 and Control 2) from the actual EAG values. Correlation data shows relative abundance of 15 blend components and their corresponding mean absolute EAG values. Details of data analyses were indicated in the main text and Figure 1 legend. Raw data behind the representation shown in Figure 1 and analyses referred to in the Results section are included. DOI: 10.5256/f1000research.10104.d143446 ^17^.
