# Supplementary material for: Electroantennogram response of the parasitoid, Microplitis croceipes to host-related odors: The discrepancy between relative abundance and level of antennal responses to volatile compound
Source: F1000Res. 2017 Mar 9;5:2725. Originally published 2016 Nov 21. [Version 2] doi: 10.12688/f1000research.10104.2 (PMC5302146; doi:10.12688/f1000research.10104.2)
Supplement: Peer review history

# Peer review: Referee response for version 2

**DOI:** 10.5256/f1000research.11957.r20837

**Published:** 2017-04-11

**Authors:** Torsten Meiners

**Affiliations:** 1 Federal Research Centre for Cultivated Plants Ecological Chemistry, Plant Analysis and Stored Product Protection, Julius Kühn-Institut (JKI), Berlin, Germany

The authors provided more information on a previous study and on the methods in the revision. They furthermore introduced the research topic and discussed their results more comprehensively.

I have read this submission. I believe that I have an appropriate level of expertise to confirm that it is of an acceptable scientific standard.

# Peer review: Referee response for version 1

**DOI:** 10.5256/f1000research.10885.r19829

**Published:** 2017-02-13

**Authors:** Feng Liu

**Affiliations:** 1 Department of Entomology, Michigan State University, East Lansing, MI, USA

**General comments**

 This study investigated the EAG response of a parasitoid *Microplitis croceipes* to the volatiles emitted from its host, the cotton plant-fed *Heliothis virescens* larvae. 15 compounds were tested at the dose of 1 μg on the female antennae of *M. croceipes*. Different level of bioactivity of *M. croceipes* to these compounds were presented and discrepancy was observed between the relative abundance and level of antennal responses in parasitoids. In the end, the authors suggested that ecological relevance but not the relative abundance of these compounds weighted more on the EAG responses of *M. croceipes*. However, since there is no behavior bioassay showing that these host-released compounds were truly importantly in the host-seeking process, it is hard to tell the ecological relevance of those compounds (like decanal) with strong EAG responses. A more cautious conclusion would be appropriate.

**Specific comments**

 Introduction: Please give more information about why the authors specifically stated that the**** *Heliothis virescens* larvae were cotten-fed in the lab. Will different food sources affect the compounds released from the insect bodies?

 Method: Please justify why mass concentration was used in preparing the compounds. Different chemicals possess various molecular weight and vapor pressure. Therefore, the number of molecules delivered onto the antenna may be dramatically different. In addition, only female wasps were used in the experiments. Apparently females need to find host to lay eggs. Just curious to know what the male's EAG responses to these compounds or if there are any related studies. 

 Results: Since 50% of the EAG responses from blend volatiles were used as a standard to make comparison, it would be better to add the EAG response to the blend volatiles in the bar figure. In addition, please specify the EAG response to the control solvent (hexane).

 Discussion: The authors initiated a good start to discuss some compounds in the blend may function as background odors to enhance olfactory contract. There are many excellent reviews about the possible mechanisms behind this pheromone, such as Riffell and Hildebrand (2016).The authors may discuss a little bit about the mechanisms.

I have read this submission. I believe that I have an appropriate level of expertise to confirm that it is of an acceptable scientific standard.

# Peer review: Referee response for version 1

**DOI:** 10.5256/f1000research.10885.r19230

**Published:** 2017-02-10

**Authors:** Torsten Meiners

**Affiliations:** 1 Federal Research Centre for Cultivated Plants Ecological Chemistry, Plant Analysis and Stored Product Protection, Julius Kühn-Institut (JKI), Berlin, Germany

The research note of Tolulope et al. reports EAG responses of the parasitic wasp *Microplitis croceipes *and relates the antennal responses to the relative abundance of the compounds in the odour bouquet of the larvae.

 When looking at the relative abundance of a compound you might need to consider all environmentally occurring contexts and compounds. *Microplitis *is orientating to the host habitat first, then it locates the host plant in the habitat, and then the host. That means that plant odours play an important role (see Li et al. ^1^) and not only larval odours. In my opinion you have to rank the importance of odours according to all environmental contexts and consider the abundance of compounds in cotton or also in other relevant host (and habitat) plants. *Heliothis* feeds on more than 100 plant species, thus *M. croceipes *is confronted with the odour of this plants and with the odour of larvae having fed on these plants. Li et al. ^1^ have performed antennal studies with *M. croceipes* and cotton plant compounds and found similar responses to similar compounds as in your study. Heptanal was the most stimulating tested compound while caryophyllene was less stimulating. The the discrepancy you indicate in the title might be easily explained when including habitat and host plant volatiles.

 The wasps in your study had the experience of living and hatching from larvae having fed on cotton. Thus they might have experience with the compounds you present in Table 1. It has been shown that *M. croceipes* can learn almost any compund (e.g. Olson et al. ^2^).

 In your discussion you point out that it might be the ecological relevance of a compound that determines the antennal response – however, Park et al. ^3^ showed in electroantennogram studies that the antenna of *M. croceipes *is also responding to anthropogenic compounds with high sensitivity. Thus, the antennal response might not reflect the ecological relevance. This might be more reflected in the behavioural response, as you indicate in your discussion. And this might be fine-tuned by leaning in case of a parasitoid with a polyphagous host.

 Minor points:

 Methods: Why is 1 µg an optimal dose?

 Data analyses:  Differences …were analysed

I have read this submission. I believe that I have an appropriate level of expertise to confirm that it is of an acceptable scientific standard, however I have significant reservations, as outlined above.

## References

[1] LiYDickensJCSteinerWW: Antennal olfactory responsiveness ofMicroplitis croceipes (Hymenoptera: Braconidae) to cotton plant volatiles. *J Chem Ecol*.1992;18(10) : 10.1007/BF02751101 1761-73 10.1007/BF02751101 24254718

[2] OlsonDMRainsGCMeinersTTakasuKTertulianoMTumlinsonJHWäckersFLLewisWJ: Parasitic wasps learn and report diverse chemicals with unique conditionable behaviors. *Chem Senses*.2003;28(6) :545-9 1290759210.1093/chemse/28.6.545

[3] ParkKZhuJHarrisJOchiengSBakerT: Electroantennogram responses of a parasitic wasp, Microplitis croceipes, to host-related volatile and anthropogenic compounds. *Physiological Entomology*.2008;26(1) : 10.1111/j.1365-3032.2001.00219.x 69-77 10.1111/j.1365-3032.2001.00219.x

# Peer review: Referee response for version 1

**DOI:** 10.5256/f1000research.10885.r19925

**Published:** 2017-02-06

**Authors:** Yonggen Lou

**Affiliations:** 1 National Key Laboratory of Rice Biology, Institute of Insect Science, Zhejiang University, Hangzhou, China

The manuscript reported the the relationship between the relative abundance of volatile chemical emitted from *Heliothis virescens *larvae and the antennal response by the larval parasitoid of the herbivore, *Microplitis croceipes*. By electroantennogram bioassays, the authors found that the level of parasitoid’s EAG response is not related to relative abundance of the volatile components in the blend. Since a chemical that elicits an EAG response in an insect does not mean to elicit a behavioral response, and a chemical eliciting a bigger EAG response does not mean to elicit a stronger behavioral response, these experiments seem little and the novelty and significance of this study seem limited.

 Introduction: In a previous study, the authors have investigated the attractiveness of these individual volatile compounds to the parasitoid, thus it would be better to introduce these results briefly in the section of Introduction.

 Methods: It has been well documented that an insect has different responsive ranges to different chemicals. Thus, only using one concentration of chemicals is not enough.

 Discussion: Based on the previous results reported by the authors ^1^, 8 chemicals in the blend, including  decanal and ( *E*)-β-Caryophyllene, had a role in attraction of the parasitoid. The authors should give a discussion based on above results.

I have read this submission. I believe that I have an appropriate level of expertise to confirm that it is of an acceptable scientific standard.

## References

[1] MorawoTFadamiroH: Identification of Key Plant-Associated Volatiles Emitted by Heliothis virescens Larvae that Attract the Parasitoid, Microplitis croceipes: Implications for Parasitoid Perception of Odor Blends. *J Chem Ecol*.2016;42(11) : 10.1007/s10886-016-0779-7 1112-1121 10.1007/s10886-016-0779-7 27722877

# Peer review: Referee response for version 1

**DOI:** 10.5256/f1000research.10885.r19376

**Published:** 2017-01-16

**Authors:** Amanuel Tamiru

**Affiliations:** 1 International Centre of Insect Physiology and Ecology - ICIPE , Nairobi, Kenya

**General comments:**

 This study examined the relationship between the relative abundance of a volatile organic compounds (VOCs) emitted by *Heliothis virescens* (Lepidoptera: Noctuidae) and level of antennal response by the larval parasitoid, *Microplitis croceipes* (Hymenoptera: Braconidae). The study builds on the previous work by Morawo & Fadamiro (2016) which identified key plant-associated volatiles emitted by cotton-fed *H. virescens* larvae that attract the parasitoid, *M. croceipes. *Here, the synthetic versions of 15 previously identified plant-associated volatiles emitted by *H. virescens* larvae were tested in electroantennogram (EAG) bioassays. The authors conclude that the level of parasitoid’s antennal response is not directly related to relative abundance of the volatile components rather influenced by the ecological relevance or functional role of the compound in the blend. It should be noted that identifying volatile compounds that insects detect through EAG is an initial step in understanding olfactory stimuli responsible for modulating insect behavior. Hence, behavioral studies with the identified EAG active compounds should be carried out, individually and as blends, to determine responses of parasitoids to the volatile compounds and explore their ecological or functional role. Though the authors intended to examine relationship between relative abundance of the VOCs and level of antennal responses, different levels of the volatile compounds used in the study and their corresponding EAG response is not shown. Rather, only one dose (1 µg) is used for all volatile compounds tested. The study would have been very informative if different levels of the test compounds are tested and their corresponding EAG response recorded.

**Specific comments**

**Title:** The title is appropriate for the content of the article; however, it can be made more concise. E.g. the first part of the existing title would adequate, i.e. ‘Electroantennogram response of the parasitoid, *Microplitis croceipes *to host-related odors’.

**Introduction: **The introduction clearly states the objective of the study. However, adequate background is missing in the area of herbivore emitted plant associated VOCs. Are these plant derived volatile compounds emitted by herbivore itself (after feeding) or are they adsorbed into the hervivore body during feeding process (e.g. from frass)

**Methods and materials: **The authors followed standard insect rearing (Lewis & Burton, 1970) and electroantennogram recording (Ngumbi *et al.*, 2010) and data analysis procedures. However, the number of insect replicates used in the study is not clear. Was a single insect antennal preparation used to record EAG responses to the 15 compounds and control (hexane)? Was the abundance of volatile components varied based on the corresponding quantities in the natural headspace samples? The latter has not been specified in the methodology except mentioning ‘ *Test compounds were formulated in hexane at 0.1 μg/μl and delivered onto Whatman®No.1 filter paper strips at an optimum dose of 1 µg*’.

**Results:** The results show that female *M. croceipes* showed varying EAG responses to test compounds. Notably, decanal which constituted only 1% of total blend emitted by cotton-fed *H. virescens* elicited the highest EAG response (0.82 mV); while ( *E*)-β-caryophyllene, the most abundant component (29.2% of total blend), elicited a relatively low EAG response (0.17 mV) in the parasitoid antenna. This is possible as earlier reports also indicated compounds with highest EAG response may not necessarily be those emitted in largest quantity (Tamiru *et al.* 2015 ^1^). Given the fact that the main research question of this study is examine the relationship between relative abundance and level of antennal responses, it would have been more informative to test different concentrations/amounts of the test compounds and their corresponding EAG response to reliably measure statistical significance of correlations between relative abundance of the compounds and level of EAG response.

**Discussion:** The authors discuss about the ecological relevance of a compounds. However, electrophysiological responses do not necessarily mean that a behavioral response will occur; rather it elucidates potential behaviorally relevant compounds. To fully explore the significance of the results, bioassays need to be carried out with identified compounds, both individually and as a blend, to determine the kind of behavioral response the volatile compounds trigger in the parasitoid and their ecological/functional role. I suggested authors to refer and perhaps include in their discussion the work by Tamiru et al. (2015) ^1^ which demonstrated combined use of electrophysiological and behavioral studies for better understanding of odor mediated behavior in insects.

I have read this submission. I believe that I have an appropriate level of expertise to confirm that it is of an acceptable scientific standard.

## References

[1] TamiruABruceTWoodcockCBirkettMMidegaCPickettJKhanZ: Chemical cues modulating electrophysiological and behavioural responses in the parasitic waspCotesia sesamiae. *Canadian Journal of Zoology*.2015;93(4) : 10.1139/cjz-2014-0266 281-287 10.1139/cjz-2014-0266
